# Factors associated with repeated refusal to participate in longitudinal population-based HIV surveillance in rural South Africa: an observational study, regression analyses

**Published:** 2012

**Authors:** Katie Giordano, Till Bärnighausen, Nuala McGrath, Rachel Snow, Siobán Harlow, Marie-Louise Newell

**Affiliations:** 1 Department of Epidemiology, School of Public Health, University of Michigan, Ann Arbor, USA; 2 Department of Health Behavior & Health Education, School of Public Health, University of Michigan, Ann Arbor, USA; 3 Africa Centre for Health and Population Studies, University of KwaZulu-Natal, Somkhele, South Africa; 4 Department of Global Health and Population, Harvard School of Public Health, Harvard University, Boston, USA; 5 London School of Hygiene and Tropical Medicine, London, UK; 6 Centre for Paediatric Epidemiology and Biostatistics, University College London Institute of Child Health, UK

## Abstract

**Background:**

For many estimation purposes, individuals who repeatedly refuse to participate in longitudinal HIV surveillance pose a bigger threat to valid inferences than individuals who participate at least occasionally. We investigate the determinants of repeated refusal to consent to HIV testing in a population-based longitudinal surveillance in rural South Africa.

**Methods:**

We used data from two years (2005 & 2006) of the annual HIV surveillance conducted by the Africa Centre for Health and Population Studies, linking the HIV surveillance data to demographic and socioeconomic data. The outcome for the analysis was “repeated refusal”. Demographic variables included sex, age, highest educational attainment, and place of residence. We also included a measure of wealth and the variable “ever had sex”. To compare the association of each variable with the outcome, unadjusted odds ratios and standard errors were estimated. Multivariable logistic regression was used to estimate adjusted odds ratios and their standard errors. Data were analyzed using STATA 10.0.

**Results:**

Of 15,557 eligible individuals, 46% refused to test for HIV in both rounds. Males were significantly more likely than females to repeatedly refuse testing. Holding all other variables constant, individuals in the middle age groups were more likely to repeatedly refuse testing compared with younger and older age groups. The odds of repeated refusal increased with increasing level of education and relative wealth. People living in urban areas were significantly more likely to repeatedly refuse an HIV test than people living in peri-urban or rural areas. Compared to those who had ever had sex, both males and females who had not yet had sex were significantly more likely to refuse to participate.

**Conclusions:**

The likelihood of repeated refusal to test for HIV in this longitudinal surveillance increases with education, wealth, urbanization, and primary sexual abstinence. Since the factors determining repeated HIV testing refusal are likely associated with HIV status, it is critical that selection effects are controlled for in the analysis of HIV surveillance data. Interventions to increase consent to HIV testing should consider targeting the relatively well educated and wealthy, people in urban areas, and individuals who have not yet sexually debuted.

## OBJECTIVES

Data from population-based longitudinal HIV surveillances can be used to estimate levels of HIV prevalence and incidence [1, 2], investigate factors associated with positive HIV status and HIV acquisition [3, 4], and monitor the impact of prevention interventions and antiretroviral treatment. Although participation in longitudinal HIV surveillance may be imperfect, potentially leading to biased inferences, for many purposes it suffices if individuals eligible to participate in the surveillance test in some but not all of the surveillance rounds. For instance, to estimate the total number of people in need of HIV care at one point in time, it suffices if all eligible HIV-infected individuals tested once before the time of estimation. In contrast, if a proportion of HIV-infected individuals never participated in HIV testing, estimates of the number of people needing HIV care are likely to be biased. To design interventions to improve longitudinal HIV surveillance, it is thus of prime importance to understand the determinants of repeated refusal to participate in HIV testing.

A number of studies have investigated factors associated with refusal to test for HIV in voluntary counseling and testing (VCT) service settings, showing that self-reported sexual risk behaviour, education, socioeconomic status, and urban residence are associated with the likelihood of testing [5–10]. The VCT setting, however, differs in many dimensions from longitudinal population-based surveillance. Individuals actively seek out testing in a VCT facility, concerned about their individual HIV risk and prepared to receive information. In contrast, in a population-based HIV surveillance individuals are approached in their homes, and participation in the surveillance serves a collective purpose, leading to information about the development of the HIV epidemic in the community. A few studies have investigated factors associated with testing in longitudinal HIV surveillance [11–16] but none have examined the determinants of repeated non-participation. We investigate for the first time the determinants of repeated refusal to participate in HIV testing in a longitudinal population-based HIV surveillance in rural Africa.

## METHODS

### Setting and surveillance operations

We used data from the annual HIV surveillance conducted by the Africa Centre for Health and Population Studies (AC), University of KwaZulu-Natal. The AC was established to provide high quality data to monitor the rapidly progressing HIV epidemic in South Africa and to evaluate interventions. The data collected by the AC Demographic Information System (ACDIS) is available in a single database, which allows linkage of a wide range of variables at individual, household and community levels. We linked the HIV surveillance data to demographic and socioeconomic data on individuals eligible to participate in HIV testing [4]. In the surveillance, demographic information (collected every 6 months) and socioeconomic data (collected once per year) are elicited from household proxy respondents, i.e., on household members’ reports on all other household members (e.g., education level) and household-level variables (e.g., assets). The data from the demographic surveillance is used to construct the eligibility list for the HIV and behaviour surveillance, which is conducted on different days than the demographic and socioeconomic surveillance. In the HIV and behaviour surveillance, each individual who is resident in the household at the last demographic surveillance visit and meets the age criteria (15–49 years of age, for females, and 15–54 years for males) is eligible to participate in HIV testing and to respond to the behaviour questionnaire. Individuals can refuse to answer any of the survey questions, but unlike HIV testing the surveillance staff does not elicit consent to the participation in the survey interviews. For a detailed description of ACDIS, see the AC website [17] and Tanser et al. [18]. At the time of this study (2005 and 2006), the results of HIV tests conducted as part of the surveillance were available on-demand to participants two weeks after the fieldworkers visit in one of 16 HIV voluntary counseling and testing (VCT) centres operated by the AC. HIV test results could be accessed through handheld computers operated by trained HIV counselors after entry of a confidential pin number held by the individual surveillance participants. During the HIV surveillance fieldworker visits and during VCT centre visits, all contacted individuals were informed that CD4 count testing and HIV antiretroviral treatment (ART) were available free of charge at the public-sector primary-care clinics in the demographic surveillance area (DSA) and the wider district. Since the start of the public-sector ART scale-up in South Africa in late 2004, the AC has partnered with the Department of Health in the delivery of ART through the Hlabisa sub-district HIV Treatment and Care Programme. The AC contribution to the Programme has been supported by the United States Agency for International Development (USAID) and the President’s Emergency Program for AIDS Relief (PEPFAR). Ethics permission for the demographic and HIV surveillance was obtained from the University of KwaZulu-Natal Bio-medical Research Ethics Committee.

The DSA is located in KwaZulu-Natal, South Africa, near the small-town of Mtubatuba and covers 438 km^2^ in the district of Umkanyakude. The DSA covers approximately 90,000 resident and non-resident members of roughly 11,000 households. The area is predominantly rural, but includes an urban township and peri-urban informal settlements, as is typical of many rural areas of South Africa [18]. Information from the household surveys is used to create the eligibility list for the HIV surveillance, which is drawn up at the beginning of each year and includes all resident members of households who are 15–49 (females) or 15–54 (males) years of age on the date the list is generated [4]. (Since 2007, the upper age limit in the HIV surveillance has been lifted [19].) Teams of trained field workers, one male and one female, visit each household, attempting to contact each individual in his/her home, in up to 4 attempts. No other criteria besides sex are used to match interviewer and respondent. Following written informed consent, a finger prick of blood is taken and prepared as a dried blood spot for HIV testing by ELISA [4].

This analysis used data from the second (January to December 2005) and third round (January to December 2006) of the HIV surveillance programme.

### Sample and variables

Our overall sample for analysis includes all individuals (N=15,557) who were age-eligible for inclusion in the HIV surveillance during the second and third HIV surveillance rounds and resident in the demographic surveillance area during both rounds. We choose the second and the third round of the HIV surveillance for this sample, rather than the first round, because the first round was an outlier regarding consent to participate in the surveillance, with substantially higher consent rates [18]. As reported in Tanser et al, 2% of residents could not be contacted in 2005 and 8% could not be contacted in 2006 [18]. The outcome for the current analysis was “repeated refusal”, with individuals coded “yes” if they refused to provide a sample for HIV testing in both surveillance rounds and “no”, if they agreed to provide a sample in at least one of the two rounds. Demographic variables from ACDIS included sex, age, highest educational attainment, and place of residence (urban, peri-urban, rural) [17]. We used a household assets index as a measure of wealth. Household assets indices are valid proxies for wealth in surveys in rural Africa [20]. Following Filmer and Pritchett [21], we used the first principal component obtained in a principal component analysis of information on house ownership, water source, energy, toilet type, electricity and 27 household assets as an assets index [4]. We categorized households in tertiles (poorest to wealthiest).

The sexual behaviour component of the HIV surveillance included information on the timing of sexual debut [22]. We created a variable “ever had sex”, coding any individual who reported either to have sexually debuted or to have had sex in the past year as ever having had sex.

### Statistical analysis

To compare the association of each variable with the outcome “repeated refusal”, unadjusted odds ratios (uOR) and standard errors (SE) were estimated.

Multivariable logistic regression was used to estimate adjusted odds ratio (aOR) point estimates and their SEs as a measure of sampling uncertainty around aORs (Tables 1 – 8); 95% confidence intervals around the ORs may be roughly approximated by OR ± 2 × SE. We chose modal values as reference categories. We conducted all analyses separately for males (Tables 1, 3, 5 & 7) and females (Tables 2, 4, 6 & 8) to allow the relationships between repeated refusal and all explanatory variables to vary by sex.

We explored the influence of missing values in the results of some variables by including categories for missing values for all variables in some of the analyses. Data were analyzed using STATA 10.0 (Stata Corporation., College Station, Texas, USA).

## RESULTS

Of the 15,557 eligible resident individuals contacted in both round 2 and 3 of the AC HIV surveillance, 46% refused to provide a sample for HIV testing in both rounds. Males (50% of 6419) were significantly more likely than females (44% of 9138) to refuse to provide a sample for HIV testing in both rounds (*P*<0.001).

In multivariable regression analyses of all males (Table 1) or females (Table 2) included in the overall sample, those in the middle age groups were more likely to repeatedly refuse to participate compared with the younger and older age groups (males aged 30–34 years, aOR 2.0, *P*<0.001; females aged 25–29 years, aOR 2.4, *P*<0.001). Holding all other variables constant, the odds of repeated refusal increased monotonically with increasing level of education and relative wealth. People living in urban areas within the DSA were significantly more likely to repeatedly refuse an HIV test than people living in peri-urban or rural areas (males with urban place of residence, aOR 1.6, *P*=0.004; females with urban place of residence, aOR 2.3, *P*<0.001).

Compared to those who had ever had sex, both males and females who had not yet had sex were significantly more likely to refuse to participate (males who had never had sex, aOR 1.2, *P*=0.008; females who had never had sex, aOR 1.3, *P*=0.005). In further analyses (not shown) we found that the change in the estimated relationship between “ever had sex” and repeated refusal to participate, which we observe when comparing uOR and aOR, was mostly explained by the fact that age confounds the unadjusted relationship between “ever had sex” and repeated refusal to participate.

The relationships described above remained essentially unchanged when we restricted the samples in our regressions to those individuals who did not have any missing values for any of the explanatory variables (Tables 3 and 4), which is an indication of the robustness of the results. We also found that none of the adjusted odds ratios changed by more than 15% when we restricted the analyses to those who ever had sex. See Tables 5–8 for results.

Males (Table 1) and females (Table 2) with missing information on “ever had sex” were much more likely to repeatedly refuse to provide a sample for HIV testing compared to those who had sex (males with missing information on “ever had sex”, aOR 17.3, *P*<0.001; females with missing information on “ever had sex”, aOR 12.9, *P*<0.001). Table 9 shows the percentage of those who repeatedly refused to answer questions on the sexual behaviour health survey and repeatedly refused to test for HIV. The majority of those who refused to consent to HIV testing responded to the sexual behaviour health survey questions we used to derive the variable “ever had sex”, indicating that the factors that influence HIV testing are different than those that influence refusal to answer the questions in the survey interviews (see Table 9).

Reasons for refusal to consent to an HIV test were elicited from all individuals who refused to participate in the HIV surveillance in 2006. The pre-coded question in the survey included three response options. Out of 3648 individuals who answered the question, 61% replied with “dislikes blood being taken”, 36% replied with “knows result”, and 4% replied with “nothing can be done”. The percentages add up to just over 100% because individuals were permitted to respond with more than one answer.

As indicated in the analyses section, Tables 1 through 8 report standard errors (SE) as a measure of sampling uncertainty around the OR point estimates. 95% confidence intervals around the aORs may be roughly approximated by aOR ± 2 × SE.

## DISCUSSION

We investigated for the first time factors associated with repeated refusal to participate in a longitudinal HIV surveillance in rural South Africa.

In the setting of a population-based surveillance, in which the same individuals are asked to consent to HIV testing at different points in time, we are more concerned about people who repeatedly refuse to consent than about people who consent only sometimes. The information on factors associated with repeated refusal can inform HIV surveillance systems on how to design interventions to motivate individuals who have in the past consistently refused to participate in HIV testing to test at least on some occasions in the future[23]. Almost half (46%) of the eligible individuals repeatedly refused to consent to HIV testing. Similarly high refusal rates have been found in other HIV surveys in South Africa. For instance, in the nationally representative Nelson Mandela/Human Sciences Research Council Study of HIV/AIDS the HIV refusal rates in 2002, 2005, and 2008 were 35%, 45% and 48%, respectively [24–26]. Refusal rates in HIV surveys in sub-Saharan Africa are variable but commonly high [27]. The reasons for the particularly high refusal rates in South Africa are not well understood. We used data from a large population-based HIV surveillance in rural South Africa to elucidate the reasons for repeated refusal to consent to HIV testing. Males were significantly more likely to repeatedly refuse HIV testing than females, echoing findings from cross-sectional HIV surveys in South Africa [11, 13]. In multivariable regression we find that the odds of repeated HIV test refusal increase with education and wealth, and are higher in urban than in peri-urban and rural areas.

These associations may reflect the fact that among individuals who are neither committed nor very much opposed to HIV testing in the surveillance the socioeconomically more powerful may find it easier to refuse the offer of an HIV test than those of lower status [28]. Alternatively, it is also plausible that education, wealth and urban residence increase an individual’s ability to access HIV testing outside the surveillance [7, 8] and that those who have already tested for HIV in other settings are less likely to consent to testing in the surveillance. Finally, it is plausible that people who know their status are fearful of potential negative consequences if others learn that they are HIV-infected.

Future studies need to investigate these hypotheses and evaluate interventions aimed at increasing surveillance participation in socioeconomically advantaged groups.

Never having had sex was significantly associated with repeated refusal of HIV testing both in males and females, when controlling for age, education, wealth, and place of residence. Individuals who have abstained from sex throughout their life may not see the benefits of participating in an HIV surveillance, whose data could inform the design of HIV prevention or treatment programmes.

While the factors associated with repeated refusal in this study are similar to those found in studies of single refusal, this finding is not self-evident. It would have been plausible that the factors determining repeated refusal and occasional refusal are very different. For instance, socioeconomic status could have been a strong predictor of occasional refusal but not of repeated refusal, if people of higher socioeconomic status refuse surveillance participation because they have the power to do so. From day to day, the people of higher socioeconomic status may vary substantially in their inclination to participate in the HIV surveillance (depending on their emotional state, for instance) and they may thus sometimes participate and sometimes refuse to do so. The fact that factors determining socioeconomic status are significant predictors of repeated refusal to participate in the HIV surveillance is thus a new and important insight gained through this study.

The fact that observed socioeconomic and behavioural factors significantly affect repeated refusal in an HIV surveillance underlines the importance of correcting HIV prevalence and incidence estimates for selection bias. It is, however, important to note that controlling for selection on observed factors (such as the variables investigated in this study) may not be sufficient to ensure unbiased estimation. As a recent study in Zambia demonstrates, selection on unobserved factors can substantially bias HIV prevalence estimates and should thus be routinely controlled for in the analysis [29].

One approach to account for selection of unobserved factors is to use Heckman-type selection models, with interviewer identity as a selection variable in the estimation. This approach has been described elsewhere[29, 30].

Accurate information on the development of the HIV epidemic is crucial for the design of programmes to prevent the spread of HIV and for planning services for those who are already HIV-infected. Education about the purpose of surveillance and the potential benefits to the community may help increase participation.

## CONCLUSSION

It is unlikely that the increases of past years in the funding of HIV prevention and treatment will continue [31, 32]. In order to ensure that the limited resources for HIV interventions are used efficiently, it is crucial to evaluate the performance of existing and new interventions at the population level. HIV surveillance can crucially contribute to such evaluation. As our study demonstrates, socioeconomically advantaged groups and people who have never had sex are more likely to repeatedly refuse to test for HIV in a longitudinal surveillance. Since the factors determining repeated HIV testing refusal are likely associated with HIV status, it is critical that selection effects are controlled for in the analysis of past HIV surveillance data. For future surveillance rounds, interventions aimed at increasing consent to participation in order to reduce selection effects should target the relatively well educated and wealthy, people in urban areas, and individuals who have not yet sexually debuted.

## Figures and Tables

**Table 1:** Determinants of repeated refusal of an HIV test among all males included in the overall sample.

Age (years)	*N*	%	% repeat refusal	uOR	ˆ SE	*P*	aOR	ˆ SE	*P*
15–19*	2741	43	40	1			1		
20–24	1024	16	50	1.5	(0.11)	<0.001	1.4	(0.12)	<0.001
25–29	527	8	57	1.9	(0.19)	<0.001	1.6	(0.18)	<0.001
30–34	496	8	62	2.4	(0.24)	<0.001	2.0	(0.24)	<0.001
35–39	482	7	62	2.4	(0.25)	<0.001	1.7	(0.21)	<0.001
40–44	440	7	62	2.4	(0.25)	<0.001	1.6	(0.22)	<0.001
45–49	442	7	56	1.9	(0.20)	<0.001	1.2	(0.17)	0.112
50–54 (Males only)	267	4	51	1.6	(0.20)	0.001	0.9	(0.15)	0.385
**Education level**									
No schooling	343	5	50	1.0	(0.12)	0.806	0.7	(0.11)	0.040
Primary education	1340	21	41	0.7	(0.04)	<0.001	0.7	(0.05)	<0.001
Secondary education*	3844	60	49	1			1		
> Secondary education	145	2	76	3.21	(0.63)	<0.001	1.6	(0.36)	0.045
Missing	747	12	61	1.6	(0.13)	<0.001	0.9	(0.12)	0.278
**Wealth tertiles**									
Poorest third*	1922	30	42	1			1		
Middle third	1938	30	46	1.2	(0.07)	0.026	1.1	(0.08)	0.060
Wealthiest third	2013	31	56	1.7	(0.11)	<0.001	1.3	(0.10)	0.002
Missing	546	9	65	2.5	(0.25)	<0.001	1.5	(0.25)	0.010
**Place of residence**									
Rural**	3908	61	47	1			1		
Peri-urban	2124	33	49	1.1	(0.06)	0.030	1.0	(0.07)	0.735
Urban	387	6	82	5.2	(0.71)	<0.001	1.6	(0.27)	0.004
**Ever Had Sex**									
Yes*	3590	56	39	1			1		
No	1499	23	37	0.9	(0.06)	0.232	1.2	(0.10)	0.008
Missing	1330	21	92	17.8	(1.90)	<0.001	17.3	(1.91)	<0.001

Total *N*	6419		LR chi^2^	1531.57			Prob>chi^2^		<0.001

All males in the overall sample are included in the analyses.

* Reference group, uOR/aOR = unadjusted/adjusted odds ratio, SE = standard error, LR= likelihood ratio.

ˆ 95% confidence intervals around the ORs may be roughly approximated by OR ± 2 × SE.

**Table 2:** Determinants of repeated refusal of an HIV test among all females included in the overall sample.

Age (years)	*N*	%	% repeated refusal	uOR	ˆSE	*P*	aOR	ˆSE	*P*
15–19*	2631	29	34	1			1		
20–24	1369	15	42	1.4	(0.10)	<0.001	1.4	(0.11)	<0.001
25–29	999	11	54	2.3	(0.17)	<0.001	2.4	(0.21)	<0.001
30–34	976	10	53	2.2	(0.17)	<0.001	2.2	(0.20)	<0.001
35–39	1086	12	51	2.0	(0.15)	<0.001	1.9	(0.18)	<0.001
40–44	1189	13	48	1.8	(0.13)	<0.001	1.8	(0.17)	<0.001
45–49	888	10	44	1.5	(0.12)	<0.001	1.4	(0.16)	0.001
50–54 (Males only)									
**Education level**									
No schooling	666	7	37	0.78	(0.07)	0.004	0.7	(0.07)	<0.001
Primary education	1896	21	38	0.79	(0.04)	<0.001	0.7	(0.05)	<0.001
Secondary education*	5298	58	43	1			1		
> Secondary education	293	3	77	4.5	(0.64)	<0.001	1.7	(0.28)	0.003
Missing	985	11	56	1.7	(0.12)	<0.001	0.8	(0.09)	0.029
**Wealth tertiles**									
Poorest third*	2846	31	35	1			1		
Middle third	2830	31	40	1.2	(0.07)	<0.001	1.2	(0.07)	0.006
Wealthiest third	2753	30	53	2.1	(0.12)	<0.001	1.6	(0.11)	<0.001
Missing	709	8	62	3.1	(0.27)	<0.001	2.0	(0.27)	<0.001
**Place of residence**									
Rural*	5778	63	40	1			1		
Peri-urban	2783	31	44	1.2	(0.06)	<0.001	0.9	(0.05)	0.190
Urban	577	6	84	7.8	(0.90)	<0.001	2.3	(0.31)	<0.001
**Ever Had Sex**									
Yes*	6389	70	37	1			1		
No	1414	15	33	0.9	(0.05)	0.009	1.3	(0.10)	0.005
Missing	1335	15	90	15.0	(1.41)	<0.001	12.9	(1.25)	<0.001
Total *N*	9138								

LR chi^2^							1819.39		
Prob>chi^2^							<0.001		

All females in the overall sample are included in the analyses.

* Reference group, uOR/aOR = unadjusted/adjusted odds ratio, SE = standard error, LR= likelihood ratio.

95% confidence intervals around the ORs may be roughly approximated by OR ± 2 × SE.

**Table 3:** Determinants of repeated refusal of an HIV test among males with no missing data on any variable.

Age (years)	*N*	%	% repeated refusal	uOR	ˆ SE	*P*	aOR	ˆ SE	*P*
15–19*	2294	50	34	1			1		
20–24	790	17	42	1.4	(0.12)	<0.001	1.4	(0.13)	<0.001
25–29	358	8	46	1.7	(0.19)	<0.001	1.8	(0.22)	<0.001
30–34	302	7	52	2.1	(0.26)	<0.001	2.3	(0.30)	<0.001
35–39	251	5	41	1.3	(0.18)	0.035	1.6	(0.24)	0.001
40–44	212	5	39	1.2	(0.18)	0.206	1.5	(0.25)	0.007
45–49	227	5	34	1.0	(0.14)	0.896	1.3	(0.21)	0.118
50–54 (Males only)	131	3	26	0.7	(0.14)	0.050	0.9	(0.21)	0.760
**Education level**									
No schooling	230	5	32	0.7	(0.10)	0.012	0.7	(0.12)	0.046
Primary education	1094	24	30	0.6	(0.05)	<0.001	0.7	(0.05)	<0.001
Secondary education*	3160	69	41	1			1		
> Secondary education	81	2	58	2.02	(0.46)	0.002	1.4	(0.35)	0.125
**Wealth tertiles**									
Poorest third*	1546	34	34	1			1		
Middle third	1574	34	37	1.2	(0.09)	0.052	1.1	(0.09)	0.163
Wealthiest third	1445	32	43	1.5	(0.11)	<0.001	1.3	(0.11)	0.003
**Place of residence**									
Rural*	2892	63	37	1			1		
Peri-urban	1573	35	40	1.1	(0.07)	0.055	1.0	(0.07)	0.694
Urban	100	2	54	2.0	(0.41)	0.001	1.4	(0.31)	0.113
**Ever Had Sex**									
Yes*	3161	69	38	1			1		
No	1404	31	37	0.9	(0.06)	0.408	1.3	(0.10)	0.004
Total *N*	4565								

LR chi^2^							129.06		
Prob>chi^2^							<0.001		

Males with missing values on any explanatory variable are excluded from the analyses.

* Reference group, uOR/aOR = unadjusted/adjusted odds ratio, SE = standard error, LR= likelihood ratio.

ˆ 95% confidence intervals around the ORs may be roughly approximated by OR ± 2 × SE.

**Table 4:** Determinants of repeated refusal of an HIV test among females with no missing data on any variable.

Age (years)	*N*	%	% repeated refusal	uOR	ˆ SE	*P*	aOR	ˆ SE	*P*
15–19*	2280	32	29	1			1		
20–24	1116	16	36	1.3	(0.10)	<0.001	1.5	(0.13)	<0.001
25–29	775	11	47	2.2	(0.18)	<0.001	2.5	(0.24)	<0.001
30–34	709	10	44	1.9	(0.17)	<0.001	2.2	(0.22)	<0.001
35–39	735	11	40	1.6	(0.14)	<0.001	2.1	(0.22)	<0.001
40–44	812	12	36	1.3	(0.12)	0.001	1.8	(0.19)	<0.001
45–49	600	8	31	1.1	(0.11)	0.343	1.6	(0.19)	<0.001
50–54 (Males only)									
**Education level**									
No schooling	558	8	31	0.8	(0.07)	0.005	0.7	(0.08)	0.009
Primary education	1658	24	31	0.8	(0.05)	<0.001	0.7	(0.05)	<0.001
Secondary education*	4654	66	37	1			1		
> Secondary education	157	2	63	2.9	(0.48)	<0.001	1.8	(0.32)	0.001
**Wealth tertiles**									
Poorest third*	2478	35	30	1			1		
Middle third	2428	35	35	1.2	(0.08)	0.001	1.2	(0.08)	0.003
Wealthiest third	2121	30	44	1.8	(0.11)	<0.001	1.6	(0.11)	<0.001
**Place of residence**									
Rural*	4694	67	34	1			1		
Peri-urban	2146	30	36	1.1	(0.06)	0.200	0.9	(0.05)	0.032
Urban	187	3	68	4.1	(0.66)	<0.001	2.5	(0.43)	<0.001
**Ever Had Sex**									
Yes*	5705	81	36	1			1		
No	1322	19	33	0.9	(0.06)	0.033	1.3	(0.11)	0.001
Totals *N*	7027								

LR chi^2^							301.55		
Prob>chi^2^							<0.001		

Females with missing values on any explanatory variable are excluded from the analyses.

* Reference group, uOR/aOR = unadjusted/adjusted odds ratio, SE = standard error, LR= likelihood ratio.

ˆ 95% confidence intervals around the ORs may be roughly approximated by OR ± 2 × SE.

**Table 5:** Determinants of repeated refusal of an HIV test among males.

Age (years)	aOR	ˆ SE	*P*
15–19*	1		
20–24	1.3	(0.13)	0.004
25–29	1.7	(0.19)	<0.001
30–34	2.0	(0.24)	<0.001
35–39	1.7	(0.22)	<0.001
40–44	1.6	(0.22)	0.001
45–49	1.2	(0.17)	0.167
50–54 (Males only)	0.8	(0.15)	0.323
**Education level**			
No schooling	0.8	(0.13)	0.157
Primary education	0.7	(0.06)	<0.001
Secondary education*	1		
> Secondary education	1.7	(0.42)	0.033
Missing	0.9	(0.13)	0.552
**Wealth tertiles**			
Poorest third*	1		
Middle third	1.2	(0.11)	0.021
Wealthiest third	1.4	(0.13)	0.001
Missing	1.6	(0.28)	0.014
**Place of residence**			
Rural*	1		
Peri-urban	1.0	(0.08)	0.614
Urban	1.7	(0.33)	0.008
Total *N*	4920		

LR chi^2^			1399.27
Prob>chi^2^			<0.001

Males in the overall sample are included; males who never had sex are excluded from the analyses.

* Reference group, aOR = adjusted odds ratio, SE = standard error, LR= likelihood ratio, Prob = probability;

ˆ 95% confidence intervals around the ORs may be roughly approximated by OR ± 2 × SE.

**Table 6:** Determinants of repeated refusal of an HIV test among females.

Age (years)	aOR	ˆ SE	*P*
15–19*	1		
20–24	1.5	(0.13)	<0.001
25–29	2.4	(0.22)	<0.001
30–34	2.2	(0.21)	<0.001
35–39	1.9	(0.19)	<0.001
40–44	1.7	(0.17)	<0.001
45–49	1.4	(0.16)	0.001
50–54 (Males only)			
**Education level**			
No schooling	0.7	(0.08)	0.002
Primary education	0.8	(0.06)	<0.001
Secondary education*	1		
> Secondary education	1.8	(0.31)	0.001
Missing	0.8	(0.09)	0.064
**Wealth tertiles**			
Poorest third*	1		
Middle third	1.2	(0.08)	0.003
Wealthiest third	1.6	(0.12)	<0.001
Missing	2.2	(0.31)	<0.001
**Place of residence**			
Rural*	1		
Peri-urban	0.9	(0.05)	0.143
Urban	2.0	(0.29)	<0.001
Total *N*	7724		

LR chi^2^			1702.47
Prob>chi^2^			<0.001

Females in the overall sample are included; females who never had sex are excluded from the analyses.

* Reference group, aOR = adjusted odds ratio, SE = standard error, LR = likelihood ratio, Prob = probability

ˆ 95% confidence intervals around the ORs may be roughly approximated by OR ± 2 × SE.

**Table 7:** Determinants of repeated refusal of an HIV test among males.

Age (years)	aOR	ˆ SE	*P*
15–19*	1		
20–24	1.4	(0.15)	0.001
25–29	1.9	(0.24)	<0.001
30–34	2.3	(0.31)	<0.001
35–39	1.6	(0.25)	0.001
40–44	1.5	(0.25)	0.013
45–49	1.3	(0.21)	0.158
50–54 (Males only)	0.9	(0.21)	0.695
**Education level**			
No schooling	0.8	(0.14)	0.208
Primary education	0.7	(0.07)	<0.001
Secondary education*	1		
> Secondary education	1.6	(0.41)	0.089
**Wealth tertiles**			
Poorest third*	1		
Middle third	1.2	(0.11)	0.063
Wealthiest third	1.4	(0.15)	<0.001
**Place of residence**			
Rural*	1		
Peri-urban	1.0	(0.08)	0.640
Urban	1.4	(0.40)	0.186
Total *N*	3161		

LR chi^2^			116.37
Prob>chi^2^			<0.001

Males with missing values on any explanatory variable and those who never had sex are excluded from the analyses.

* Reference group, aOR = adjusted odds ratio, SE = standard error, LR = likelihood ratio, Prob = probability

ˆ 95% confidence intervals around the ORs may be roughly approximated by OR ± 2 × SE.

**Table 8:** Determinants of repeated refusal of an HIV test among females.

Age (years)	aOR	ˆ SE	*P*
15–19*	1		
20–24	1.6	(0.15)	<0.001
25–29	2.5	(0.26)	<0.001
30–34	2.2	(0.24)	<0.001
35–39	2.1	(0.23)	<0.001
40–44	1.8	(0.20)	<0.001
45–49	1.6	(0.20)	<0.001
50–54 (Males only)			
**Education level**			
No schooling	0.8	(0.09)	0.032
Primary education	0.7	(0.06)	<0.001
Secondary education*	1		
> Secondary education	2.0	(0.37)	<0.001
**Wealth tertiles**			
Poorest third*	1		
Middle third	1.2	(0.09)	0.002
Wealthiest third	1.6	(0.13)	<0.001
**Place of residence**			
Rural*	1		
Peri-urban	0.9	(0.06)	0.033
Urban	2.2	(0.42)	<0.001
Total *N*	5705		

LR chi^2^			267.42
Prob>chi^2^			<0.001

Females with missing values on any explanatory variable and those who never had sex are excluded from the analyses.

* Reference group, aOR = adjusted odds ratio, SE = standard error, LR = likelihood ratio, Prob = probability

ˆ 95% confidence intervals around the ORs may be roughly approximated by OR ± 2 × SE.

**Table 9:** Cross-tabulation of respondents’ repeated refusal to consent to HIV testing and repeated refusal to answer the sexual behaviour question on whether they had “ever had sex”

“Ever had sex” data			
Refuse to test	Missing %	Available %	Total %
Yes	15.5	31.0	46.5
No	1.5	52.0	53.5
Total	17.0	83.0	100.0

Chi square	2.6E+03		
Prob	<0.001		

Total number of individuals: 15,557

Prob = probability

## List of abbreviations

HIV: Human immunodeficiency virus
AC: Africa Centre
ACDIS: Africa Centre Demographic Information System
VCT: Voluntary Counseling and Testing
DSA: Demographic Surveillance Area
uOR: Unadjusted odds ratio
aOR: Adjusted odds ratio
SE: Standard error
LR: Likelihood ratio
